# A biomarker framework for cardiac aging: the Aging Biomarker Consortium consensus statement

**DOI:** 10.1093/lifemedi/lnad035

**Published:** 2023-09-27

**Authors:** Aging Biomarker Consortium, Weiwei Zhang, Yang Che, Xiaoqiang Tang, Siqi Chen, Moshi Song, Li Wang, Ai-Jun Sun, Hou-Zao Chen, Ming Xu, Miao Wang, Jun Pu, Zijian Li, Junjie Xiao, Chun-Mei Cao, Yan Zhang, Yao Lu, Yingxin Zhao, Yan-Jiang Wang, Cuntai Zhang, Tao Shen, Weiqi Zhang, Ling Tao, Jing Qu, Yi-Da Tang, Guang-Hui Liu, Gang Pei, Jian Li, Feng Cao

**Affiliations:** Department of Cardiology, The Second Medical Centre, Chinese PLA General Hospital, National Clinical Research Center for Geriatric Diseases, Beijing 100853, China; State Key Laboratory of Cardiovascular Disease, Fuwai Hospital, National Center for Cardiovascular Diseases, Chinese Academy of Medical Sciences and Peking Union Medical College, Beijing 100037, China; Key Laboratory of Birth Defects and Related Diseases of Women and Children of MOE, State Key Laboratory of Biotherapy, West China Second University Hospital, Sichuan University, Chengdu 610041, China; State Key Laboratory of Membrane Biology, Institute of Zoology, Chinese Academy of Sciences, Beijing 100101, China; University of Chinese Academy of Sciences, Beijing 100049, China; State Key Laboratory of Membrane Biology, Institute of Zoology, Chinese Academy of Sciences, Beijing 100101, China; University of Chinese Academy of Sciences, Beijing 100049, China; Beijing Institute for Stem Cell and Regenerative Medicine, Beijing 100101, China; Institute for Stem Cell and Regeneration, Chinese Academy of Sciences, Beijing 100101, China; State Key Laboratory of Cardiovascular Disease, Fuwai Hospital, National Center for Cardiovascular Diseases, Chinese Academy of Medical Sciences and Peking Union Medical College, Beijing 100037, China; Department of Cardiology, Zhongshan Hospital, Fudan University, Shanghai Institute of Cardiovascular Diseases, Shanghai 200433, China; Key Laboratory of Viral Heart Diseases, National Health Commission, Shanghai 200433, China; Key Laboratory of Viral Heart Diseases, Chinese Academy of Medical Sciences, Shanghai 200433, China; Institutes of Biomedical Sciences, Fudan University, Shanghai 200032, China; Department of Biochemistry and Molecular Biology, State Key Laboratory of Medical Molecular Biology, Institute of Basic Medical Sciences, Chinese Academy of Medical Sciences and Peking Union Medical College, Beijing 100005, China; Department of Cardiology and Institute of Vascular Medicine, Peking University Third Hospital, Beijing 100191, China; NHC Key Laboratory of Cardiovascular Molecular Biology and Regulatory Peptides, State Key Laboratory of Vascular Homeostasis and Remodeling, Peking University, Beijing 100191, China; State Key Laboratory of Cardiovascular Disease, Fuwai Hospital, National Center for Cardiovascular Diseases, Chinese Academy of Medical Sciences and Peking Union Medical College, Beijing 100037, China; Clinical Pharmacology Center, Fuwai Hospital, National Center for Cardiovascular Diseases, Chinese Academy of Medical Sciences and Peking Union Medical College, Beijing 100037, China; State Key Laboratory for Oncogenes and Related Genes, Department of Cardiology, Shanghai Jiao Tong University, Shanghai 200025, China; Department of Cardiology and Institute of Vascular Medicine, Peking University Third Hospital, Beijing 100191, China; Beijing Key Laboratory of Cardiovascular Receptors Research, Key Laboratory of Cardiovascular Molecular Biology and Regulatory Peptides, State Key Laboratory of Vascular Homeostasis and Remodeling, Peking University, Beijing 100191, China; Department of Pharmacy, Peking University Third Hospital, Beijing 100191, China; Shanghai Engineering Research Center of Organ Repair, School of Medicine, Shanghai University, Shanghai 200444, China; Cardiac Regeneration and Ageing Lab, Institute of Cardiovascular Sciences, School of Life Science, Shanghai University, Shanghai 200444, China; Laboratory of Cardiovascular Science, Beijing Clinical Research Institute, Beijing Friendship Hospital, Capital Medical University, Beijing 100050, China; Capital Institute of Pediatrics, Beijing 100020, China; Graduate School of Peking Union Medical College, Beijing 100730, China; State Key Laboratory of Membrane Biology, Institute of Molecular Medicine, College of Future Technology, Peking University, Beijing 100871, China; Institute of Cardiovascular Sciences and Key Laboratory of Molecular Cardiovascular Sciences, School of Basic Medical Sciences, Ministry of Education, Peking University Health Science Center, Beijing 100191, China; Clinical Research Center, The Third Xiangya Hospital, Central South University, Changsha 410013, China; Department of Cardiology, Beijing Anzhen Hospital, Capital Medical University, Beijing Institute of Heart Lung and Blood Vessel Disease, Beijing 100029, China; Department of Neurology and Centre for Clinical Neuroscience, Daping Hospital, Third Military Medical University, Chongqing 400042, China; Institute of Brain and Intelligence, Third Military Medical University, Chongqing 400042, China; Chongqing Key Laboratory of Ageing and Brain Diseases, Chongqing 400016, China; Center for Excellence in Brain Science and Intelligence Technology, Chinese Academy of Sciences, Shanghai 200031, China; Gerontology Center of Hubei Province, Wuhan 430000, China; Institute of Gerontology, Department of Geriatrics, Tongji Hospital, Tongji Medical College, Huazhong University of Science and Technology, Wuhan 430030, China; The Key Laboratory of Geriatrics, Beijing Institute of Geriatrics, Institute of Geriatric Medicine, Chinese Academy of Medical Sciences, Beijing Hospital/National Center of Gerontology of National Health Commission, Beijing 100730, China; University of Chinese Academy of Sciences, Beijing 100049, China; Institute for Stem Cell and Regeneration, Chinese Academy of Sciences, Beijing 100101, China; CAS Key Laboratory of Genomic and Precision Medicine, Beijing Institute of Genomics, Chinese Academy of Sciences and China National Center for Bioinformation, Beijing 100101, China; Department of Cardiology, Xijing Hospital, the Fourth Military Medical University, Xi’an 710032, China; University of Chinese Academy of Sciences, Beijing 100049, China; Beijing Institute for Stem Cell and Regenerative Medicine, Beijing 100101, China; Institute for Stem Cell and Regeneration, Chinese Academy of Sciences, Beijing 100101, China; State Key Laboratory of Stem Cell and Reproductive Biology, Institute of Zoology, Chinese Academy of Sciences, Beijing 100101, China; Department of Cardiology and Institute of Vascular Medicine, Peking University Third Hospital, Key Laboratory of Molecular Cardiovascular Sciences (Peking University), Ministry of Education, NHC Key Laboratory of Cardiovascular Molecular Biology and Regulatory Peptides, Key Laboratory of Cardiovascular Receptors Research, Beijing 100191, China; State Key Laboratory of Membrane Biology, Institute of Zoology, Chinese Academy of Sciences, Beijing 100101, China; University of Chinese Academy of Sciences, Beijing 100049, China; Beijing Institute for Stem Cell and Regenerative Medicine, Beijing 100101, China; Institute for Stem Cell and Regeneration, Chinese Academy of Sciences, Beijing 100101, China; Advanced Innovation Center for Human Brain Protection, and National Clinical Research Center for Geriatric Disorders, Xuanwu Hospital Capital Medical University, Beijing 100053, China; Shanghai Key Laboratory of Signaling and Disease Research, Laboratory of Receptor-Based Biomedicine, The Collaborative Innovation Center for Brain Science, School of Life Sciences and Technology, Tongji University, Shanghai 200070, China; The Key Laboratory of Geriatrics, Beijing Institute of Geriatrics, Institute of Geriatric Medicine, Chinese Academy of Medical Sciences, Beijing Hospital/National Center of Gerontology of National Health Commission, Beijing 100730, China; Department of Cardiology, The Second Medical Centre, Chinese PLA General Hospital, National Clinical Research Center for Geriatric Diseases, Beijing 100853, China

## Abstract

Cardiac aging constitutes a significant risk factor for cardiovascular diseases prevalent among the elderly population. Urgent attention is required to prioritize preventive and management strategies for age-related cardiovascular conditions to safeguard the well-being of elderly individuals. In response to this critical challenge, the Aging Biomarker Consortium (ABC) of China has formulated an expert consensus on cardiac aging biomarkers. This consensus draws upon the latest scientific literature and clinical expertise to provide a comprehensive assessment of biomarkers associated with cardiac aging. Furthermore, it presents a standardized methodology for characterizing biomarkers across three dimensions: functional, structural, and humoral. The functional dimension encompasses a broad spectrum of markers that reflect diastolic and systolic functions, sinus node pacing, neuroendocrine secretion, coronary microcirculation, and cardiac metabolism. The structural domain emphasizes imaging markers relevant to concentric cardiac remodeling, coronary artery calcification, and epicardial fat deposition. The humoral aspect underscores various systemic (*N*) and heart-specific (*X*) markers, including endocrine hormones, cytokines, and other plasma metabolites. The ABC’s primary objective is to establish a robust foundation for assessing cardiac aging, thereby furnishing a dependable reference for clinical applications and future research endeavors. This aims to contribute significantly to the enhancement of cardiovascular health and overall well-being among elderly individuals.

## Introduction

Aging gracefully is a common aspiration, yet it is accompanied by not only wisdom but also the onset of diseases. With the global elderly population continuing to grow at an unprecedented rate, the substantial prevalence of age-related diseases has imposed a considerable social and economic burden in China and worldwide [1]. The degenerative alterations in both the structure and function of the heart that accompany aging constitute significant risk factors for various cardiovascular diseases. In turn, the occurrence of cardiovascular diseases accelerates the aging process of the heart. Hence, the early detection of cardiac degeneration, timely warnings, preventive measures, and treatment of cardiovascular diseases hold paramount importance in the pursuit of healthy aging [2].

Cardiac aging encompasses a series of structural remodeling and functional changes within the heart that manifest with advancing age [3]. Principal phenotypic changes include a decrease in left ventricular volume, an increase in the ventricular mass-to-volume ratio, insufficiencies of chordae tendineae and papillary muscles, valve stenosis and insufficiency, coronary artery obstructions and calcifications, epicardial fat deposition, heightened cardiac fibrosis, left ventricular diastolic dysfunction, impaired cardiac conduction function, and cardiac neuroendocrine dysfunction. The search for biomarkers capable of reflecting the degree of cardiac aging is pivotal for accurately assessing the cardiac functional status and predicting the risk of age-related cardiac diseases. This pursuit, in turn, facilitates tailored interventions aimed at disease prevention and management.

On 5 August 2023, the Aging Biomarker Consortium (ABC) convened a symposium in Beijing, bringing together professionals from the field of cardiac aging [4]. Drawing upon the latest domestic and international scientific breakthroughs, an expert consensus, incorporating clinical evidence and expert opinions, has been established. The recommended biomarkers have the capacity to address pressing clinical inquiries, including “What is the biological age of an individual’s heart?”, “How rapidly is an individual’s heart aging?”, and “How close is an individual to age-related cardiac disease?”.

## Methods

We conducted comprehensive literature searches encompassing studies published prior to June 2023, utilizing well-known databases such as MEDLINE, PubMed, the Cochrane Library, and other pertinent sources germane to this consensus. For a detailed account of the specific search terms employed, readers are encouraged to consult the online data supplement, which includes the final evidence tables that summarize the information utilized by the consensus writing group to formulate the recommendations.

Based on the amalgamation of available publications and collective research, the members of the ABC collaboratively identified a compendium of fundamental issues concerning cardiac aging biomarkers through online collaboration. Subsequently, the identified biomarkers underwent comprehensive deliberation during an in-person meeting to achieve a unanimous consensus. All recommendations underwent meticulous scrutiny and discussion among the ABC members, thereby encompassing multi-dimensional perspectives and considerations that are elaborated in this consensus document. We have adhered to internationally accepted conventions for expressing the level of evidence and the strength of recommendations, as delineated in Table 1 [5].

**Table 1.** Class of recommendations and level of evidence

Class (strength) of recommendation	Level (quality) of evidence
Class I (Strong) Benefit >>> Risk	Level A
Suggested phrases for writing recommendation Recommended/indicated Evidence and/or general agreement that a given treatment or procedure is beneficial, useful, and effective	Data derived from multiple randomized clinical trials or meta-analyses
Class IIa (Moderate) Benefit >> Risk	Level B
Suggested phrases for writing recommendation Should be considered Weight of evidence/opinion is in favor of usefulness/efficacy	Data derived from a single randomized clinical trial or large non-randomized studies
Class IIb (Weak) Benefit ≥ Risk	Level C
Suggested phrases for writing recommendation May be considered Usefulness/efficacy is less well established by evidence/opinion	Consensus of expert opinion, and/or small studies, retrospective studies, registries
Class III (Strong) Risk > Benefit	Note: COR and LOE are determined independently (any COR may be paired with any LOE). COR, class of recommendation; LOE, level of evidence.
Suggested phrases for writing recommendation Not recommended Evidence/general agreement that the given treatment/procedure is not useful/effective and sometimes maybe harmful	

## Classification and clinical application of cardiac aging biomarkers

**Figure 1.**
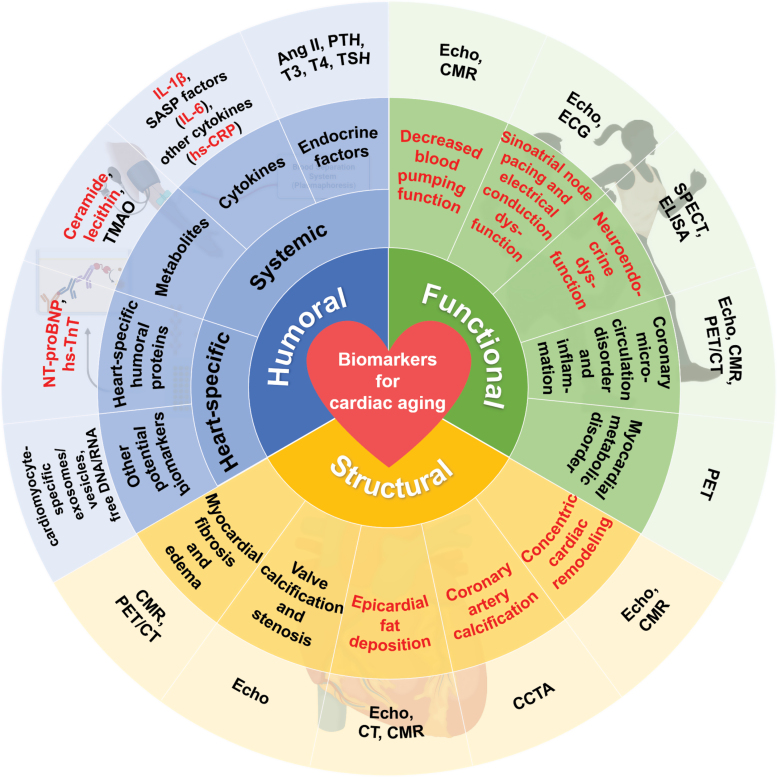
**Biomarker framework for cardiac aging.** The proposed framework for assessing cardiac aging comprises three dimensions: functional, structural, and humoral biomarkers. The most strongly recommended cardiac aging biomarkers, highlighted in red, encompass a wide range of changes occurring at various levels within the heart during the aging process. These biomarkers hold the potential for widespread utilization in routine clinical practice. However, it is essential to emphasize that further validation is required to assess their effectiveness in evaluating biological cardiac aging. Abbreviations: Echo, echocardiography; CMR, cardiac magnetic resonance imaging; ECG, electrocardiograph; SPECT, single photon emission computed tomography; ELISA, enzyme-linked immunosorbent assay; PET, positron emission tomography; CT, computed tomography; CCTA, coronary computed tomography angiography; Ang II, angiotensin II; PTH, parathyroid hormone; T3, triiodothyronine; T4, thyroxine; TSH, thyroid stimulating hormone; IL-1β, interleukin-1β; IL-6, interleukin-6; hs-CRP, high-sensitivity C-reactive protein; TMAO, trimethylamine N-oxide; NT-proBNP, N-terminal pro-B-type natriuretic peptide; hs-TnT, hypersensitive troponin T.

Cardiac aging encompasses multi-dimensional and multi-level changes occurring in molecules, cells, organs, organisms, and populations. Cardiac aging biomarkers denote indicators capable of accurately predicting the “actual age of the heart,” its structural integrity, and functional capacity. These biomarkers serve the purpose of gauging the extent of cardiac aging and evaluating the effectiveness of interventions aimed at mitigating its effects. Given the considerations of clinical feasibility and convenience, this consensus delineates the screening of cardiac aging markers across three dimensions: cardiac function, cardiac structure, and humoral factors. These findings serve as a valuable reference for clinical management and future investigations (Fig. 1; Table 2).

**Table 2.** Biomarker recommendations for cardiac aging

Dimension	Biomarker	Testing method	COR	LOE
Functional markers	Decreased diastolic function	Echo, CMR	I	B
	Decreased systolic function	Echo, CMR	IIa	B
	Sinus node pacing and electrical conduction dysfunction	Echo, ECG	IIa	B
	Abnormal cardiac neuroendocrine function	SPECT imaging, ELISA for endocrine factors	IIa	B
	Disturbance of coronary microcirculation and inflammation	Echo, CMR, PET/CT imaging	IIb	B
	Myocardial metabolic dysfunction	PET imaging	IIb	C
Structural markers	Concentric cardiac remodeling	Echo, CMR	I	B
	Coronary artery calcification	CCTA	IIa	B
	Epicardial fat deposition	Echo, CT imaging, CMR	IIa	B
Humoral markers	Non-heart-specific plasma markers IL-1β, IL-6, hs-CRP, ceramide, and lecithin	Plasma/ELISA	IIa	B
	Endocrine factors (Ang II, PTH, T3, T4, and TSH), cytokines (GDF15, CCL17, and IGFBP7), and small metabolic molecules (TMAO)	Plasma/ELISA	IIa	B
	IL-18, PAI1, TGF-β, MMPs, EDNs, and other factors	Plasma/ELISA	IIb	C
	Heart-specific plasma markers NT-proBNP, hs-TnT	Plasma/ELISA	IIa	B

Abbreviations: Echo, echocardiography; CMR, cardiac magnetic resonance imaging; ECG, electrocardiograph; SPECT, single photon emission computed tomography; ELISA, enzyme-linked immunosorbent assay; PET, positron emission tomography; CT, computed tomography; CCTA, coronary computed tomography angiography; IL-1β, interleukin-1β; IL-6, interleukin-6; hs-CRP, high-sensitivity C-reactive protein; Ang II, angiotensin II; PTH, parathyroid hormone; T3, triiodothyronine; T4, thyroxine; TSH, thyroid stimulating hormone; GDF15, growth differentiation factor 15; CCL17, C–C motif chemokine ligand 17; IGFBP7, insulin-like growth factor-binding protein-7; TMAO, trimethylamine N-oxide; IL-18, interleukin-18; PAI1, plasminogen activator inhibitor-1; TGF-β, transforming growth factor-β; MMPs, matrix metalloproteinases; EDNs, endothelins; NT-proBNP, N-terminal pro-B-type natriuretic peptide; hs-TnT, hypersensitive troponin T.

### Functional characteristics and assessment of cardiac aging

The heart functions as the vital powerhouse of the human circulatory system, often referred to as the “life pump” responsible for sustaining the normal physiological processes of various organs. Through rhythmic contractions, the heart continuously pumps blood supplying oxygen and nutrients to the body while eliminating metabolic waste products. This essential process ensures the body’s overall well-being. However, aging is accompanied by alterations in cardiac function, primarily characterized by a decline in pumping efficiency, disruptions in sinus node pacing and electrical conduction, and perturbations in neuroendocrine activity. Furthermore, cardiac aging is accompanied by phenomena such as coronary inflammation, microcirculatory disturbances, and anomalous myocardial metabolism. The functional characteristics of cardiac aging endorsed by this consensus encompass the age-related changes in cardiac function that have been identified through diverse technological approaches in prior research.

#### Decreased blood pumping function

The heart, a muscular, hollow organ, orchestrates the movement of blood. Through successive contractions and relaxations, it guides venous blood back to the right atrium and subsequently propels it into the right ventricle. Following pulmonary circulation and gas exchange, oxygenated blood enters the left atrium before being propelled into the arteries via the left ventricle, ensuring systemic circulation. Each heartbeat corresponds to a mechanical cycle of contraction and relaxation known as the cardiac cycle. The rhythmic contractions and relaxations of the atria and ventricles provide the driving force for the heart’s normative pumping function. With advancing age, both systolic and diastolic functions of the heart deteriorate, resulting in aberrations in cardiac pumping efficiency [6].

##### Decreased systolic function

In clinical practice, the assessment of cardiac systolic function frequently relies on indices such as ejection fraction (EF), stroke volume (SV), global longitudinal strain (GLS), and heart rate (HR).

At rest, there are no significant age-related alterations in cardiac EF, SV, or HR, and cardiac output remains preserved [7, 8]. However, when employing more sensitive parameters like GLS and longitudinal strain rate of the left ventricle, researchers have discerned a decline in the longitudinal systolic function of the heart with advancing age. Conversely, the left ventricular torsion index (TMI) exhibits an increase, potentially serving as a compensatory mechanism to uphold left ventricular ejection fraction during the aging process [9]. An echocardiography study involving 1105 subjects from the Atherosclerosis Risk in Communities study (ARIC) revealed a decrease in left ventricular longitudinal strain (0.39% ± 0.19%, *P* = 0.038) and an increase in the left ventricular torsion index (0.33° ± 0.04°, *P* < 0.001) with advancing age [10].

During exercise, the alterations in stroke output with age remain less conclusive. Nevertheless, both the maximum HR achievable and the EF attainable by the heart significantly diminish. Furthermore, the chronotropic response to exercise diminishes, signifying that aging results in a reduction in cardiac reserve capacity [7, 8, 11]. In a study encompassing 5,437 asymptomatic women, with an average follow-up period of 15.9 ± 2.2 years, it was observed that the peak HR achieved during exercise decreased with age (*r* = －0.62, *P* < 0.0001) [11]. Another study involving 200 subjects investigated the impact of age on cardiac function and demonstrated that the maximum EF attainable during exercise decreased with age (*β* = －0.18, *P* = 0.0001) among healthy individuals who underwent rigorous screening to exclude potential coronary artery disease [12]. In a separate study, 722 elderly subjects were examined twice every 4 years, revealing a decline in systolic function associated with systemic arteriosclerosis, reduced aortic and peripheral vascular elasticity, decreased compliance, and increased cardiac load [13].

Moreover, the physiological aging process entails a chronic and progressive deterioration of ventricular–arterial coupling. In clinical practice, the measurement of effective arterial elasticity (*E*_a_) and left ventricular end-systolic elasticity (*E*_lv_) can be accomplished through cardiac ultrasound or cardiac magnetic resonance imaging (CMR). The *E*_a_ to *E*_lv_ ratio provides insight into the degree of ventricular–arterial coupling. In the Baltimore Longitudinal Study on Aging, which involved 129 subjects aged 21–96 years, cardiac magnetic resonance testing revealed that the rate of *E*_a_ increase over time (*β* = 0.50, *P* ≤ 0.0001) surpasses the rate of *E*_lv_ increase over time (*β* = 0.35, *P* ≤ 0.001). Additionally, the study identified an upward trend in the *E*_a_/*E*_lv_ value over time (*β* = 0.25, *P* = 0.0001), signifying ventricular–arterial decoupling [14].

Collectively, these findings underscore the absence of a significant decline in systolic function at rest, with the maintenance of cardiac output. Nevertheless, more sensitive evaluative parameters like GLS and TMI are requisite for the assessment of cardiac systolic function. The pronounced reduction in cardiac reserve capacity during exercise indicates the potential of age-related cardiac decline. Peak HR and maximum EF during exercise emerge as potential biomarkers of cardiac aging, while impaired ventricular–arterial coupling can be assessed through the *E*_a_/*E*_lv_ ratio.

##### Decrease in left ventricular diastolic function

In the context of heart aging, the diminishing left ventricular diastolic function emerges as an earlier and more pronounced feature than systolic function decline [15]. Normally, left ventricular diastolic filling encompasses two stages: early diastolic passive filling and late diastolic atrial systolic active filling. With advancing age, the rate of passive filling during early diastole decelerates with the majority of ventricular filling occurring during late diastole.

In clinical practice, the evaluation of left ventricular diastolic function entails the measurement of various parameters, including early diastolic peak velocity (*E*), late diastolic peak velocity (*A*), early diastolic peak velocity (*e*ʹ) at the root of the mitral annulus, the *E*/*A* ratio, the *E*/*e*ʹ ratio, and others, utilizing cardiac ultrasound or CMR. Additionally, assessment may also encompass indicators such as early diastolic longitudinal displacement peak, early diastolic longitudinal displacement velocity, late diastolic longitudinal displacement peak, and others employing CMR [16].

A foreign study involving 35 subjects revealed that the early diastolic filling rate of the left ventricle progressively decelerates after the age of 20, resulting in an average 50% reduction by the age of 80 [17]. Although early diastolic filling in the left ventricle slows during early diastole, a higher proportion of filling transpires during late diastole, leading to an intensified *A* wave [17, 18]. Under normal circumstances, the *E*/*A* ratio exceeds 1. However, as individuals age, ventricular hypertrophy and diminished ventricular wall elasticity contribute to elevated early diastolic ventricular pressure. Consequently, this culminates in a reduction in the early diastolic ventricular filling rate and an augmented contribution of atrial contraction to left ventricular filling. As a result, the *E*/*A* ratio gradually declines [19, 20]. Another overseas study involving 2524 subjects enrolled in the ARIC demonstrated a decline in *e*ʹ (0.6 ± 1.4 cm/s, *P* < 0.001) and an increase in *E*/*e*ʹ (3.1 ± 4.4, *P* < 0.001) after a follow-up period of 6.6 ± 0.8 years [21].

Furthermore, a study conducted on 2876 healthy subjects at the First Affiliated Hospital of China Medical University unveiled significantly lower *E*/*A* (0.96 ± 0.34 vs. 1.28 ± 0.37, *P* = 0.0001) and *e*ʹ values (11.47 ± 2.64 cm/s vs. 12.91 ± 2.51 cm/s, *P* = 0.0001) among elderly subjects compared to their younger counterparts. The composite biological age score, which incorporates eight variables including *E*/*A* and *e*ʹ, can gauge the rate of heart aging in healthy Chinese individuals [22]. In accordance with a cardiac ultrasound study involving 1763 individuals at varying risk levels for heart disease, *e*ʹ values exhibited a decline with age (9.7 ± 1.7 cm/s vs. 5.1 ± 1.6 cm/s, *P* < 0.001), with the decline reaching its zenith later in life. The reduction in *e*ʹ emerges as the earliest cardiac degenerative change associated with cardiac risk factors [23].

The aforementioned results underscore the capacity of diastolic function changes to accurately mirror the aging status of the heart, thus serving as potential markers of heart aging. The assessment of cardiac diastolic function may be conducted through echocardiography employing the *E*/*A* or *E*/*e*ʹ ratio.

#### Sinoatrial node pacing and electrical conduction dysfunction

In the orchestration of systemic blood circulation, the heart’s muscular contractions necessitate synchronized electrical excitation propagation between cells. This vital conduction of excitation relies on localized currents and commences at the sinoatrial node, the heart’s natural pacemaker [9]. Throughout the aging process of the heart, the sinoatrial node, atrioventricular node, and Purkinje system undergo degenerative alterations. These changes can impede the conduction of action potentials, disrupt the electrical conductivity of heart tissue, extend conduction times, and increase ectopic beats [16].

Sinus node dysfunction exerts an impact on the heart’s pacing function, often leading to sinus bradycardia. Compared to younger, healthy individuals, the autonomy of the sinoatrial node in the elderly diminishes, along with weakened control over other conduction points. This is evident in the reduction of both maximum HR and intrinsic HR, making sinus bradycardia more prevalent [24, 25]. Moreover, abnormal electrocardiographic function in the elderly can contribute to ectopic pacing rhythms (arrhythmias), such as atrial fibrillation and atrial premature beats, which become more common after the age of 65 [26]. Pacing and functional degradation of the electrical conduction system, including sinus bradycardia and cardiac conduction block, frequently manifest as weakness, fatigue, and, in severe cases, may culminate in syncope and sudden cardiac death [27].

In clinical practice, the assessment of sinoatrial node pacing and electrical conduction function relies on pulse and electrocardiogram (ECG) evaluations. A slow pulse, particularly a slow and irregular pulse, or the absence of an increase in pulse rate during exercise, signifies sinus node dysfunction. Compared to younger individuals, the ECG of elderly subjects predominantly exhibits widened *P* waves, prolonged *P*–*R* and *Q*–*T* intervals, diminished QRS and *T* wave voltage, and a leftward shift in the QRS axis [28]. Additionally, cardiac ultrasound tissue Doppler imaging (TDI) permits the measurement of PA-TDI, which entails calculating the duration from the onset of the *P* wave to the peak of tissue Doppler *A*ʹ on the electrocardiogram. This measurement can be employed to predict heart’s electrical conduction function. One study involving 386 subjects with unstructured heart disease demonstrated that age growth was independently associated with PA-TDI prolongation (*β* = 0.476, *P* < 0.0001) [29].

Pacing and electrical conduction dysfunction exhibit a strong correlation with cardiac aging, thus signifying their potential as markers of heart aging.

#### Neuroendocrine dysfunction

The heart serves not only as a vital blood circulation pump but also plays a pivotal role in endocrine function. Cardiomyocytes possess the capacity to produce an array of hormones and bioactive substances, including atrial natriuretic peptide, antiarrhythmic peptide, and myocardial growth factor, which regulate the normal functioning of the cardiovascular system [30, 31]. However, aging individuals often experience abnormal neuroendocrine function characterized by excessive secretion of angiotensin II (Ang II), endothelin (EDN), and norepinephrine. Furthermore, there is a decrease in the clearance rate of norepinephrine, leading to abnormal neuroendocrine function within the cardiovascular system [32, 33].

In clinical practice, the evaluation of cardiac adrenergic neuron function can be achieved through ^123^I-labeled Iodine-123-meta-Iodobenzylguanidine (^123^I-MIBG) SPECT imaging. A study with a limited sample size revealed a significant negative correlation between age and the heart’s uptake rate of ^123^I-MIBG (*r* = −0.6264, *P* < 0.001) [34]. Another study demonstrated that aging can alter the heart’s response to β-adrenergic stimulation. Specifically, compared to young subjects, elderly individuals exhibited a 66% higher average concentration of norepinephrine (*P* < 0.05) and a 22% lower plasma clearance rate of norepinephrine (*P* < 0.05) [35].

While cardiac neuroendocrine dysfunction is linked to cardiac aging, its correlation is stronger with specific diseases, and there is currently no clearly defined clinical index for its assessment. Thus, its evaluation necessitates a comprehensive approach combined with other indices.

#### Coronary microcirculation disorder and inflammation

Coronary microcirculation encompasses the system of microcirculation composed of arterioles, myocardial capillaries, and venules within the heart. The coronary artery plays a crucial role in regulating blood flow to the heart and supplying oxygen to cardiomyocytes [36]. However, during the aging process, the coronary arteries within myocardial tissue undergo remodeling, leading to the abnormal functioning of coronary endothelial cells. Consequently, this results in coronary microcirculation dysfunction and inflammation [37].

Coronary flow reserve (CFR) offers insight into the adaptability of epicardial vessels and microcirculation to increased cardiac workloads. Aging is associated with a decline in CFR. Clinically, the CFR value, signifying the ratio of congested coronary blood flow to resting coronary blood flow, can be measured through various methods such as cardiac ultrasound, myocardial contrast, CMR, or positron emission tomography (PET) following intravenous administration of vasodilators like adenosine. A small cross-sectional study employing echocardiography to measure CFR in patients without abnormal myocardial perfusion found a correlation between increasing age and impaired CFR (*β* = −0.48, *P* < 0.001). The primary cause of this impairment was a reduction in blood flow velocity in the congested coronary artery due to aging (*β* = −0.30, *P* = 0.008) [38]. In addition, an echocardiographic study involving 335 subjects with normal coronary angiography indicated a gradual decline in CFR with age (first quartile 3.01 ± 0.69, fourth quartile 2.39 ± 0.49, *P* < 0.0001). This decline was primarily attributed to an increase in resting coronary blood flow velocity with age (first quartile 26.3 ± 6.1 cm/s, fourth quartile 30.2 ± 6.4 cm/s, *P* < 0.0001) [39]. Uren *et al.* examined 56 healthy subjects aged 21–86 and measured resting and congested coronary blood flow using PET scanning with ^15^O-water labeling. They found that resting coronary blood flow significantly increased with age (*r* = 0.45, *P* < 0.025). However, coronary blood flow congestion decreased significantly in subjects over 70 years old [40].

The index of microcirculatory resistance (IMR), derived from pressure and temperature measurements, quantifies microcirculatory resistance and specifically reflects resistance within the coronary microcirculation. It can be measured via cardiac catheterization and offers functional evaluation of the microcirculation from the epicardial coronary artery to the myocardium [41]. To assess the impact of aging on IMR, a study conducted cardiac catheterization combined with echocardiography on 228 patients. The results showed that as individuals age, microvascular resistance in a congested state increases (*R*^2^ = 0.02, *P* = 0.003), and the difference between the resting state and congested state decreases (*R*^2^ = 0.03, *P* = 0.002) [42].

Furthermore, ^18^F-FDG-PET/CT imaging can be clinically employed to measure the target-to-background ratio (TBR) of coronary artery myocardium. This quantitative assessment can evaluate the extent of coronary artery inflammation and identify early-stage atherosclerosis. In a study of 309 elderly subjects with no prior history of coronary artery disease, a significant correlation was found between aging and an increase in coronary artery TBR (*r* = 0.18, *P* = 0.004). The elevated coronary artery TBR suggests an increased risk of cardiovascular events (HR 4.25, *P* = 0.003) [43].

The presence of coronary microcirculation disturbance and inflammation raises the possibility of heart aging, suggesting its potential as a marker for cardiac aging. Clinical quantification of these factors can be achieved through CFR, IMR, and ^18^F-FDG assessments. However, it is important to note that CFR and IMR are both invasive tests and may not be suitable for early screening.

#### Myocardial metabolic disorder

Cardiac metabolic substrate conversion is closely linked to heart aging. The acquisition of energy substrates by cardiac myocytes is vital for generating a substantial amount of ATP. However, as individuals age, the energy substrate profile of cardiac myocytes undergoes alteration, leading to aberrant myocardial metabolism and subsequent damage to cardiac function [44, 45].

In clinical practice, the rates of myocardial fatty acid utilization and myocardial glucose utilization can be assessed using PET scans labeled with ^11^C-palmitate and ^11^C-glucose. Additionally, PET can measure myocardial blood flow through ^15^O-water scanning, myocardial oxygen consumption via ^11^C-acetate, myocardial fatty acid utilization and oxidation rates with ^11^C-palmitate, and myocardial glucose utilization rates using ^11^C-glucose. A study with a limited sample size discovered that the myocardial fatty acid utilization rate (35 ± 10 nmol FFA/nmol O_2_ × 10^−3^ vs. 51 ± 20 nmol FFA/nmol O_2_ × 10^−3^, *P* < 0.005) and oxidation rate (33 ± 10 nmol FFA/nmol O_2_ × 10^−3^ vs. 48 ± 18 nmol FFA/nmol O_2_ × 10^−3^, *P* < 0.004) of elderly subjects were significantly lower than those of young subjects. However, there was no significant change in the absolute utilization rate of myocardial glucose [46].

Throughout the aging process, while the absolute value of the myocardial glucose utilization rate remains relatively stable, the relative proportion of myocardial glucose metabolism within the myocardial metabolic substrate may increase due to the decline in myocardial fatty acid utilization rate. However, the clinical significance of this metabolic transformation warrants further investigation.

##### Recommendations

(1) The decline in diastolic function serves as a significant hallmark of heart aging. Clinically, this can be assessed via echocardiography using *E*/*A* or *E*/*e*ʹ, and it is considered a reliable marker of cardiac aging (Level B evidence, Class I recommendation).
(2) Systolic function decline can indicate the extent of heart aging. Clinically, parameters like GLS, TMI, *E*_a_/*E*_lv_, peak HR after exercise loading, and maximum EF can be utilized for evaluation. These parameters are identified as potential markers of cardiac aging (Level B evidence, Class IIa recommendation).
(3) Sinus node pacing and electrical conduction dysfunction suggest the possibility of cardiac aging, increasing the likelihood of arrhythmia and atrial fibrillation. Clinical evaluation can be based on PA-TDI characteristics and dynamic ECG, including *P* wave, *T* wave, *P*–*R* interval, *Q*–*T* interval, and QRS axis. These parameters can serve as markers of cardiac aging (Level B evidence, Class IIa recommendation).
(4) Abnormal cardiac neuroendocrine function indicates the potential for cardiac aging and can be evaluated using ^123^I-MIBG and humoral markers such as endocrine factor indicators. It can be considered a functional marker of cardiac aging, but validation is needed in subsequent cohort studies (Level B evidence, Class IIa recommendation).
(5) Disorders in coronary microcirculation and inflammation often accompany cardiac aging. Indicators like ^18^F-FDG, CFR, and IMR can be considered for clinical assessment and serve as functional markers of cardiac aging (Level B evidence, Class IIb recommendation).
(6) Cardiac metabolic substrate conversion is associated with cardiac aging. Clinically, the utilization rate of myocardial fatty acids, myocardial glucose, and other indicators can be detected through PET scans employing ^11^C-palmitate and ^11^C-glucose labeling. The clinical significance of this marker of cardiac aging function necessitates further research (Level C evidence, Class IIb recommendation).

### Structural characteristics and assessment of cardiac aging

Cardiac aging encompasses a spectrum of changes spanning from cellular alterations to macroscopic structural transformations. Within clinical settings, a range of imaging techniques, including echocardiography, cardiac computed tomography (CT), CMR, and nuclear imaging, are utilized to assess the structural changes associated with cardiac aging. The structural characteristics outlined in this consensus draw from age-related cardiac structural modifications observed through diverse imaging modalities in prior research endeavors.

#### Concentric cardiac remodeling

Concentric cardiac remodeling is characterized by a reduction in the volume of the cardiac chamber accompanied by an increase in the ratio of ventricular mass to volume. This phenomenon represents one of the most common alterations in the aging heart. Age-related oxidative stress contributes to a reduction in myocardial cell count, increased extracellular matrix deposition, and compensatory hypertrophy of remaining cells, ultimately leading to a decrease in the volume of the cardiac chamber [9]. In clinical practice, parameters such as left ventricular end-diastolic volume (LVEDV), left ventricular end-systolic volume, left ventricular end-diastolic volume index (LVEDVI), and mass-to-volume ratio (M/V ratio) can be measured using echocardiography or CMR to assess concentric remodeling.

Longitudinal studies conducted by the Multi-Ethnic Study of Atherosclerosis (MESA) have demonstrated that LVEDV gradually decreases with aging, while the M/V ratio increases. However, an increase in left ventricular mass with age is observed primarily in males [47]. A study at Xuanwu Hospital involving 550 healthy subjects aged 21–70 who underwent CMR revealed that as age increases, LVEDV (*r* = −0.31, *P* < 0.001) and LVSDV (*r* = −0.37, *P* < 0.001) decrease, while end-diastolic left ventricular wall thickness (*r* = 0.31, *P* < 0.001) increases. However, the increase in left ventricular mass with age is observed only in females (*r* = 0.36, *P* < 0.001) [48].

Previous studies have highlighted the thickening of the interventricular septum along with an increase in left and right atrial volumes with age [16, 49, 50]. However, a recent CMR study involving a cohort of over 14,000 individuals has revealed a different trend. This study found that the interventricular septum initially thickens toward the left ventricle with age, followed by a subsequent decrease in thickness as age advances [50]. Additionally, another CMR study at Fuwai Hospital, involving 200 healthy subjects aged 20–70, demonstrated a significant reduction in end-systolic left atrial diameter (*r* = −0.25, *P* < 0.001) and end-systolic right atrial volume (*r* = −0.22, *P* = 0.002) with increasing age [51]. Moreover, a machine learning analysis of CMR data from the UK Biobank, encompassing 26,893 participants, revealed a significant negative correlation between age and left atrial maximum volume (LAV max) (*β* = −2.0, *P *= 9.1 × 10^−44^), right atrial maximum volume (RAV max) (*β* = −1.0, *P* = 1.7 × 10^−9^), as well as left ventricular myocardial mass (LVM) (*β* = −1.0, *P* = 2.2 × 10^−12^) [52]. In summary, these results suggest a propensity for concentric cardiac remodeling with increasing age, characterized by a reduction in left ventricular chamber volume and an increased *M*/*V* ratio. However, the relationship between aging and variables such as ventricular mass, interventricular septal thickness, and atrial dimensions remains controversial and requires further research.

#### Coronary artery calcification

Cardiac aging is associated with an increased occurrence of obstructive lesions and calcification within the coronary arteries [53]. As individuals age, the coronary artery intima undergoes processes including lipid oxidation, endothelial dysfunction, inflammatory responses, and the formation of apoptotic bodies [54]. These apoptotic bodies contain essential calcium and phosphate ions necessary for the accumulation of calcific crystals, leading to the deposition of hydroxyapatite and subsequent coronary intimal calcification. Simultaneously, aging is linked to reduced renal function and disturbances in calcium-phosphate metabolism, contributing to abnormal calcium salt deposition and coronary medial calcification [55]. Clinically, coronary computed tomography angiography (CCTA) is a valuable tool for evaluating the severity of coronary artery lumen stenosis and plaque calcification. The coronary artery calcification score (CACS), often derived using the Agatston score, is a robust indicator for assessing cardiovascular risk [56].

A sub-study of the “Progression of AtheRosclerotic PlAque DetermIned by Computed TomoGraphic Angiography Imaging” (PARADIGM) study, which included 1153 subjects undergoing continuous CCTA, revealed a notable trend. With increasing age, there is an increase in total plaque volume (first quartile 7.8 mm^3^/year, fourth quartile 12.1 mm^3^/year, *P* = 0.001) and an elevation in dense calcification volume (first quartile 2.5 mm^3^/year, fourth quartile 7.1 mm^3^/year, *P* < 0.001) [57]. A cohort study at the General Hospital of the PLA, involving 338 elderly patients and utilizing CCTA, reported a similar pattern. With advancing age, a significant increase was observed in the percentage of obstructive coronary artery lesions (*P* < 0.001), accompanied by an accelerated growth rate in the volume of calcified plaques [58]. These findings establish a correlation between the aging process and the heightened presence of coronary artery calcified plaques. The CACS, assessed through CCTA, holds potential as a marker for cardiac aging.

#### Epicardial fat deposition

Cardiac aging leads to an increased deposition of epicardial fat within the pericardium. As individuals age, chronic hypoxic damage to the epicardium becomes more prevalent, resulting in the accumulation of white adipose tissue in this region. This phenomenon is associated with the synthesis and secretion of pro-inflammatory adipokines by epicardial fat, which, in turn, induce processes such as cardiac fibrosis, atrial arrhythmias, and the progression of atherosclerosis [59]. Various imaging techniques are employed to quantify epicardial fat deposition. Echocardiography measures epicardial fat tissue thickness, cardiac CT assesses the total volume of epicardial fat tissue, and CMR allows for the quantification of the epicardial fat volume index [60].

A study involving a limited sample size of 58 healthy subjects who underwent CMR demonstrated that the epicardial fat volume index in older subjects was significantly higher than that in younger subjects (54.7 mL/m^2^ ± 27.1 vs. 34.5 mL/m^2^ ± 18.6, *P* < 0.01) [61]. Analysis of results from the MESA cohort, comprising 6,814 participants who underwent cardiac CT, revealed that epicardial fat volume index increases with age (*P* < 0.001). Among various ethnicities, Chinese males exhibited the largest epicardial fat volume (adjusted for height, age, and site) (*P* < 0.001). Furthermore, a significant correlation was found between epicardial fat volume and the severity of coronary artery calcification (PR 1.06, 95%CI 1.04–1.08, *P* < 0.0001) [62]. These findings suggest that epicardial fat deposition increases with age and that the epicardial fat volume may serve as a potential marker of cardiac aging.

#### Valve calcification and stenosis

Cardiac aging is also characterized by valve calcification and stenosis. The underlying mechanism involves age-related impairment of endothelial barrier function, which allows lipid infiltration into the subendothelial space, triggering inflammation, fibrosis, matrix remodeling, and calcium deposition, ultimately leading to valve calcification and stenosis [63]. Aortic valve stenosis, the most prevalent valvular heart disease, becomes more common with age [64]. Echocardiography is currently the gold standard for diagnosing aortic valve stenosis, with a normal aortic valve area typically ranging from 3 to 4 cm^2^. During the early stages of the disease, the aortic valve area generally decreases by 0.1 cm^2^ annually, although the exact rate varies based on individual risk factors [65].

It is important to note that not all elderly individuals may exhibit cardiac valve calcification or stenosis. Therefore, when clinically assessing cardiac aging, a comprehensive evaluation should include indicators of cardiac valve calcification and stenosis in conjunction with other markers of cardiac aging.

#### Myocardial fibrosis and edema

Cardiac aging is associated with an increase in collagen deposition and myocardial fibrosis, as well as an elevation in ventricular filling pressure, which can lead to myocardial edema [66, 67]. Clinically, CMR contrast-enhanced T1 mapping enables the quantification of the extracellular volume fraction (ECV) and cardiac T1 values, allowing for the assessment of the extent of myocardial fibrosis. CMR T2 mapping, which measures cardiac T2 values, provides a means to evaluate the severity of myocardial edema. A study involving the MESA cohort of 1231 participants suggested a correlation between advanced age and reduced cardiac *T*1 values (*P* < 0.05), along with an increase in ECV values (slope = 0.052% per year, *P* = 0.012) [50]. Furthermore, two studies with small sample sizes indicated that cardiac *T*2 values increase with age [68, 69].

Recent advancements in nuclear imaging technology have revealed that fibroblast-activating protein inhibitors (FAPI) can serve as prospective radioactive tracers for imaging fibroblast activation. ^68^Ga-FAPI and ^18^F-NOTA-FAPI PET/CT allow for the quantitative measurement of myocardial TBR, enabling the assessment of myocardial fibrosis severity [70]. A small-sample study employing ^18^F-NOTA-FAPI PET/CT indicated a significant association between age and myocardial TBR values (*P* < 0.05) [71].

These studies suggest a connection between the aging process and the occurrence of myocardial fibrosis and myocardial edema. Measurements of cardiac *T*1 value, *T*2 value, and ECV value through CMR may serve as potential markers of cardiac aging. The assessment of fibroblast activation through the application of ^18^F-NOTA-FAPI and ^68^Ga-FAPI PET/CT may also hold relevance in understanding the process of cardiac aging. However, further extensive research is needed to thoroughly explore the potential correlation between fibroblast activation and cardiac aging.

##### Recommendations

(1) Concentric cardiac remodeling can serve as an imaging marker of cardiac aging. Echocardiography and CMR are clinically employed to quantify key indicators such as LVEDV, LVEDVI, and M/V ratio for assessment (Level B, Class I).
(2) Coronary artery calcification can be used as an imaging marker of cardiac aging. The CACS, determined through CCTA, are utilized for assessment (Level B, Class IIa).
(3) Epicardial fat deposition serves as an imaging marker of cardiac aging. Indicators such as epicardial fat thickness, epicardial fat volume, and epicardial fat volume index can be measured using various imaging modalities such as echocardiography, cardiac CT, and CMR for assessment (Level B, Class IIa).

### Humoral markers

The assessment of bioactive components in blood and other bodily fluids through diagnostic analyses is a minimally invasive and highly sensitive approach, making it a valuable auxiliary method for evaluating cardiac aging. In this consensus, we aimed to recommend humoral biomarkers associated with cardiac aging. To achieve this goal, we conducted a comprehensive search for systemic (*N*) biomarkers closely correlated with various aspects of cardiac aging and heart-specific (*X*) humoral markers specifically indicative of cardiac aging. These biomarkers offer valuable additional insights for diagnosing cardiac aging and guiding clinical interventions. However, it is essential to acknowledge that some of these markers may also vary in the context of acute and chronic heart diseases, as well as during the aging of other organs, thus potentially lacking specificity to cardiac aging [72–74]. In this section, we present both systemic (*N*) and heart-specific (*X*) humoral markers based on the “*N*+*X*” logical framework.

#### Systemic biomarkers

##### Endocrine factors

###### Renin–angiotensin–aldosterone system

The Renin–angiotensin–aldosterone system (RAAS) plays a pivotal role in normal cardiac development, cardiac function homeostasis, electrolyte and fluid balance maintenance, as well as blood pressure regulation. Dysregulation of the RAAS is a significant risk factor contributing to aging and cardiac diseases. Plasma Ang II levels exhibit a notable increase in older humans and other animals [75, 76], subsequently leading to cardiac aging-associated phenotypes such as myocardial hypertrophy, myocardial fibrosis, and coronary artery remodeling [77]. Therefore, plasma Ang II can be considered a humoral biomarker of cardiac aging. It is worth noting that while Ang II levels can also be elevated in patients with hypertension, this elevation may not necessarily be indicative of early cardiac aging. Consequently, Ang II should be used in conjunction with other markers to aid in cardiac aging assessment.

###### Parathyroid hormone

Parathyroid hormone (PTH) is an alkaline, single-chain polypeptide hormone secreted by the primary cells of the parathyroid gland. PTH’s primary function is to regulate calcium and phosphorus metabolism, promoting increased blood calcium and decreased blood phosphorus levels. Plasma PTH levels exhibit a close association in elderly individuals with cardiac aging phenotypes such as left ventricular hypertrophy [78], suggesting that plasma PTH could serve as a biomarker of cardiac aging.

###### Thyroid hormones

Thyroid hormones, including triiodothyronine (T3), thyroxine (T4), and thyroid-stimulating hormone (TSH), are closely related to aging, with cardiac and hepatic specificity [79]. In elderly adults, serum T3/T4 levels have been reported to be lower than those in younger adults. However, TSH levels tend to be slightly higher in older adults compared to younger adults [80]. The activity of thyroid hormones in cardiomyocytes regulates myocardial contractility and systolic function. The absence of T3/T4 and the burden of excessive TSH in older adults can disrupt the maintenance of healthy cardiac homeostasis [81]. Therefore, thyroid hormones could be considered biomarkers when assessing cardiac aging.

##### Cytokines

###### Nod-like receptor protein family pyrin domain containing-3 (NLRP3) inflammasome, interleukin-1β (IL-1β), and IL-18β

The NLRP3 inflammasome plays a significant role in the aging of the heart at both cellular and organ levels, with its function dependent on downstream effector molecules IL-1β and IL-18. These proinflammatory cytokines are responsible for inducing cardiac aging phenotypes such as cardiac fibrosis and hypertrophy [82, 83]. Given its current clinical use to assess the risk of aging-related heart disease [84, 85], plasma IL-1β can be considered a clinical biomarker of cardiac aging.

###### Senescence-associated secretory phenotype factors

Senescence-associated secretory phenotype (SASP) factors represent a class of inflammatory cytokines secreted by senescent cells, driving organ aging and functional impairment [86]. In the aging heart, fibroblasts secrete classical SASP factors such as IL-6, tumor necrosis factor-α (TNF-α), and plasminogen activator inhibitor-1 (PAI-1). Endothelial cells secrete classical SASP factors likeIL-6, PAI-1, matrix metalloproteinase-1 (MMP-1), MMP-3, and endothelin 1 (EDN1), while cardiomyocytes secrete transforming growth factor-β2 (TGF-β2), growth differentiation factor 15 (GDF15), and other non-classical SASP factors. These SASP factors play pivotal roles in cardiac aging and disease [87]. Abundant clinical evidence supports the relationship between plasma levels of some SASP factors and cardiac aging.

TGF-β family: This family of proteins serves as an important marker of cellular and organ aging. TGF-β can induce senescence in various cell types within cardiac tissues, contributing to systemic aging and a decline in heart function [88]. For instance, activin A, a member of the TGF-β family, plays a key role in heart aging. A clinical study involving 899 participants demonstrated that aging leads to increased plasma levels of activin A, promoting age-related cardiac hypertrophy and a decline in systolic and diastolic functions [89]. Consequently, activin A may be considered a biomarker associated with cardiac aging.

Endothelin: Endothelial cells secrete a class of peptides (EDN1-3) that activate fibroblasts or induce cardiomyocyte hypertrophy, resulting in cardiac aging phenotypes like myocardial fibrosis and hypertrophy [90]. Plasma EDN levels significantly increase in the aging population [90]. A clinical study involving 1538 participants in the United States of America demonstrated that elevated plasma EDN1 levels increase the risk of age-related decreased left ventricular diastolic function [91]. Another clinical cohort study showed that plasma levels of EDN1 are associated with the risk of aging-related left ventricular remodeling [92]. Therefore, EDN1 can be considered a humoral biomarker associated with cardiac aging.

GDF15: Plasma GDF15 levels significantly increase in older individuals, increasing the risk of cardiac aging. For instance, a meta-analysis of 53,486 participants revealed that elevated plasma GDF15 levels are associated with an increased risk of age-related myocardial infarction [93]. Another 10-year follow-up study of 2001 elderly community participants demonstrated that plasma GDF15 levels are closely associated with age-related atrial fibrillation and affect 10-year all-cause mortality [94]. Thus, GDF15 can be considered a humoral biomarker associated with cardiac aging, along with other markers.

###### Other cytokines

High-sensitivity C-reactive protein (hs-CRP): This biomarker is associated with age-related pathologies, including cardiovascular disease, hypertension, diabetes, and kidney disease [95]. An observational clinical study involving 2437 participants revealed a positive correlation between plasma CRP levels and age. Furthermore, elevated plasma CRP levels are positively linked to adverse aging-related events, establishing hs-CRP as an independent marker for assessing aging-related cardiac events [84].

C-C motif chemokine ligand 17 (CCL17): This ligand promotes aging and age-related heart disease [96, 97]. Domestic and other cohort studies have consistently reported elevated plasma CCL17 levels with aging [96–98]. Higher CCL17 levels are indicative of age-related coronary heart disease and myocardial infarction [99], suggesting its potential as a humoral biomarker for assessing cardiac aging.

Insulin-like growth factor binding protein 7 (IGFBP7): Two independent clinical studies, involving 4,263 and 1,913 participants, have demonstrated significantly increased plasma levels of IGFBP7 in older individuals [98, 100]. IGFBP7 promotes cardiac aging phenotypes, including cardiac systolic dysfunction, myocardial fibrosis, and cardiac cell senescence [100]. Therefore, IGFBP7 may be considered a humoral biomarker associated with cardiac aging.

##### Metabolites

###### Ceramide and lecithin

Ceramide is a sphingomyelin implicated in inducing cardiac lipid toxicity, thereby contributing to the onset of cardiac aging and related diseases [101]. Lecithin in food also poses a significant risk factor for heart aging and related conditions [102]. Plasma ceramide and lecithin levels are closely associated with age-related adverse cardiac events. A multicenter clinical cohort study of 10,803 participants introduced the Coronary Event Risk Test 2 (CERT2), which utilizes ceramide and lecithin levels, assayed through liquid chromatography-mass spectrometry, to predict age-related coronary heart disease events [103]. Another study, focusing on an elderly cohort, demonstrated a strong correlation between CERT2 scores and cardiac aging-related events [104]. As such, the CERT2 risk score based on ceramide and lecithin emerges as a potential biomarker for evaluating cardiac aging.

###### Trimethylamine N-oxide

Trimethylamine N-oxide (TMAO) is a metabolite produced from the metabolism of lecithin found in foods. Plasma TMAO levels are significantly higher in healthy middle-aged and elderly individuals compared to younger individuals. Elevated plasma TMAO levels have been linked to age-related cardiac hypertrophy, myocardial fibrosis, atrial fibrillation, and coronary heart disease [105]. Moreover, increased TMAO levels have been associated with reduced survival in patients with heart failure [106]. A follow-up study involving 4,007 patients further revealed a close association between plasma TMAO levels and adverse cardiac aging events [102]. These findings suggest that TMAO could serve as a potential humoral biomarker of cardiac aging.

#### Heart-specific humoral biomarkers

##### Heart-specific humoral proteins

Heart-specific fluid biomarkers currently employed to diagnose cardiac function include B-type natriuretic peptide (BNP), myocardial enzyme profiles, troponin, and myoglobin. Levels of BNP and N-terminal pro-B-type natriuretic peptide (NT-proBNP) serve as crucial markers of cardiac dysfunction. A clinical study encompassing 1,079 elderly individuals identified NT-proBNP levels as robust biomarkers of biological age [107]. In addition, a cohort study involving 2,364 elderly participants demonstrated that plasma NT-proBNP levels could assess patient outcomes effectively [108]. Consequently, NT-proBNP emerges as a humoral biomarker of cardiac aging and declining cardiac function. Myocardial enzymes, highly sensitive troponin T (hs-TnT), and myoglobin also serve as vital blood biomarkers for heart damage. However, further investigation is required to determine their potential as markers of heart aging.

##### Other potential heart-specific humoral biomarkers

Other potential humoral factors that might serve as markers of heart-specific aging include cardiomyocyte-specific exosomes/vesicles and free DNA/RNA, including mRNA, microRNA, circRNA, and lncRNA, detectable in the blood [109, 110]. Additional heart-specific humoral biomarkers may be identified through a multi-omics approach [111–113]. Furthermore, cardiomyocytes also contain more mitochondria than any other type of cell. Given that mitochondrial dynamics and biogenesis have been implicated in the regulation of cardiac function and aging [114], mitochondrial-derived components, such as cell-free circulating mtDNA, may serve as potential humoral biomarkers for heart aging [115]. Although research in this area is rapidly advancing, most studies have focused on experimental animals, with limited clinical investigations [116]. The plasma concentrations of these potential humoral components and their association with aging in humans require further elucidation. Additionally, most current investigations into humoral markers of cardiac aging primarily analyze plasma components, with only a few exploring urinary factors, such as BNP [30].

###### Recommendations

(1) Several non-heart-specific plasma markers, including IL-1β, IL-6, hs-CRP, ceramide, and lecithin, have demonstrated increased levels associated with cardiac aging. These markers have found application in clinical diagnosis and prognostic analyses, rendering them suitable humoral biomarkers for evaluating cardiac aging (Level B; Class IIa).
(2) Plasma endocrine factors such as Ang II, PTH, T3, T4, and TSH, along with cytokines including GDF15, CCL17, and IGFBP7, and small metabolic molecules like TMAO, exhibit substantial clinical evidence linking them to cardiac aging. While not yet widely applied in clinical practice, these markers hold promise as humoral biomarkers for assessing cardiac aging (Level B; Class IIa).
(3) Some clinical data suggest associations between cardiac aging and plasma IL-18, PAI1, TGF-β, MMPs, and EDNs. However, their clinical application is still under investigation, making them candidate humoral biomarkers for cardiac aging (Level C; Class IIb).
(4) Classic heart-specific proteins reflecting myocardial damage, elevated plasma levels of NT-proBNP and hs-TnT indicate the risk of cardiac aging. Widely used in clinical diagnosis, they are recommended humoral biomarkers for evaluating cardiac aging (Level B; Class IIa).

### Heart age model

Over the past decades, the Framingham Risk Score has proven invaluable to clinicians in predicting the risk of coronary heart disease, utilizing traditional cardiac risk factors such as age, gender, blood pressure, total cholesterol, high-density lipoprotein, diabetes, and smoking. This foundational framework originated from the Framingham Heart Study, the world’s longest-running investigation into cardiovascular disease. However, given the multifaceted nature of cardiac aging, encompassing molecular, cellular, organ, and systemic changes, it is imperative to develop heart age evaluation models that integrate imaging, electrophysiology, molecular biology, and other interdisciplinary tools, supported by artificial intelligence modeling and data analysis techniques. To date, several models assessing heart age and predicting heart aging and related diseases have been developed and rigorously tested.

#### Heart age assessment model

Biological age has emerged as a superior metric to chronological age for gauging the extent of cardiac aging [117]. An extensive study spanning 41 countries across 6 world regions demonstrated that predicted heart age often differs significantly from chronological age, with disparities of up to 40 years [118]. Consequently, the previously mentioned cardiac aging biomarkers offer a diverse range of quantitative indicators to accurately assess the biological age of the heart, referred to as “heart age” [119–121]. However, the advent of machine learning and the establishment of comprehensive human physiological information databases have paved the way for the development of “heart age assessment models” grounded in machine learning algorithms. Presently, these models predominantly rely on data derived from CMR and ECG data [122–128]. CMR provides insights into the metabolic and anatomical characteristics of the heart, while ECG captures HR and cardiac electrical characteristics [122–126]. Although some “heart age assessment models” based on radiomics and ECG data have found application in basic and clinical research, their validation and optimization necessitate extensive validation using long-term follow-up data from large multicenter cohorts.

#### Heart aging prediction model

Numerous studies have highlighted disparities between the heart age predicted by model-based assessments and chronological age, quantified as the “heart-age gap (HAG)”. The HAG offers valuable insights into whether an individual’s heart has experienced accelerated or delayed aging, serving as a crucial reference for predicting the rate of individual heart aging and offering early warnings regarding the onset and progression of cardiac aging-related diseases. Machine learning algorithms applied to cardiac aging prediction models predominantly encompass deep neural networks, generalized linear models, gradient-boosted models, classification and regression trees, and tree-augmented naive Bayesian networks (TAN) [129–131]. Leveraging these methodologies, HAG has been employed to forecast various diseases associated with cardiac aging, including hypertension, heart failure, stroke, acute myocardial infarction, coronary artery disease, and atrial fibrillation [124, 126, 127, 132, 133]. For instance, one study identified that individuals with an HAG exceeding seven years, calculated from ECG data, exhibited a higher prevalence of preexisting cardiovascular comorbidities [134]. It is essential to recognize that due to the current lack of aging-specific biomarkers and computational models, relying solely on heart age prediction as an independent indicator of cardiac aging-related diseases is challenging. Thus, a combined approach involving disease-specific markers should be considered.

## Conclusion and future perspectives

Based on expert discussions, the following markers of cardiac aging are strongly recommended:

(1) Functional markers: These encompass diminished diastolic function, reduced systolic function, sinus node pacing, electrical conduction dysfunction, and cardiac neuroendocrine dysfunction.
(2) Structural markers: These include indicators such as concentric cardiac remodeling, coronary artery calcification, and epicardial fat deposition.
(3) Humoral markers: This category comprises non-heart-specific plasma markers (IL-1β, IL-6, hs-CRP, ceramide, and lecithin) as well as heart-specific plasma markers (NT-proBNP, hs-TnT). It is important to note that further validation of some of these markers across diverse age cohorts is warranted in the future.

The strategic roadmap for advancing cardiac aging biomarkers in China encompasses the following objectives:

(1) Establishing multi-center aging cohorts: The aim is to create and validate cardiac aging biomarkers tailored to the Chinese population through the establishment of multi-center aging cohorts within China.
(2) Defining critical stages: Identifying the crucial phases of cardiac aging and pinpointing the optimal timeframes for personalized interventions in cardiac aging and associated diseases.
(3) AI-based assessment models: Developing artificial intelligence-driven assessment models for cardiac aging and prediction models for diseases linked to cardiac aging.
(4) Facilitating collaborations: Fostering productive partnership among industry, academia, research institutions, and government bodies to facilitate translational research in the realm of cardiac aging. The ultimate objective is to enhance heart health monitoring and intervention for the elderly in China.

**Abbreviations in this consensus**

**Table:** 

	
LOE	Level of evidence
ABC	Aging Biomarker Consortium
EF	Ejection fraction
SV	Stroke volume
GLS	Global longitudinal strain
HR	Heart rate
TMI	Torsional motility index
ARIC	Atherosclerosis Risk in Communities Study
Ea	Arterial elasticity
Elv	Left ventricular end-systolic elasticity
CMR	Cardiac magnetic resonance imaging
ECG	Electrocardiograms
TDI	Tissue Doppler imaging
Ang II	Angiotensin II
EDN	Endothelin
^123^I-MIBG	Iodine-123-m-Iodobenzylguanidine
CFR	Coronary flow reserve
PET	Positron emission tomography
IMR	Index of microcirculatory resistance
TBR	Target-to-background ratio
CT	Computed tomography
LVEDV	Left ventricular end-diastolic volume
LVSDV	Left ventricular end-systolic volume
LVEDVI	Left ventricular end-diastolic volume index
M/V ratio	Mass to volume ratio
MESA	Multi-Ethnic Study of Atherosclerosis
LAV max	Left atrial maximum volume
RAV max	Right atrial maximum volume
LVM	Left ventricular myocardial mass
CCTA	Coronary computed tomography angiography
CACS	Coronary artery calcification score
PARADIGM	Progression of AtheRosclerotic PlAque DetermIned by Computed TomoGraphic Angiography Imaging
ECV	Extracellular volume fraction
FAPI	Fibroblast activating protein inhibitor
RAAS	Renin-angiotensin-aldosterone system
PTH	Parathyroid hormone
T3	Triiodothyronine
T4	Thyroxine
TSH	Thyroid-stimulating hormone
NLRP3	Nod-like receptor protein family pyrin domain containing -3
IL-1β	Interleukin-1β
IL-18	Interleukin-18
SASP	Senescence-associated secretory phenotype
TNF-α	Tumor necrosis factor-α
PAI-1	Plasminogen activator inhibitor-1
MMP-1	Matrix metalloproteinase-1
EDN1	Endothelin 1
TGF-β2	Transforming growth factor-β2
GDF15	Growth differentiation factor 15
CRP	C-reactive protein
hs-CRP	High-sensitivity C-reactive protein
CCL17	C–C motif chemokine ligand 17
IGFBP7	Insulin-like growth factor-binding protein-7
CERT2	Cardiovascular Event Risk Test 2
TMAO	Trimethylamine N-oxide
BNP	B-type natriuretic peptide
NT-proBNP	N-terminal pro-B-type natriuretic peptide
hs-TnT	Hypersensitive troponin T
FRS	Framingham risk score
HAG	Heart-age gap
DNN	Deep neural network
GLM	Generalized linear models
GBM	Gradient-boosted model
CART	Classification and regression tree
TAN	Tree-augmented naive Bayesian network
